# Increased Incidence of Herpes Zoster and Postherpetic Neuralgia in Adult Patients following Traumatic Brain Injury: A Nationwide Population-Based Study in Taiwan

**DOI:** 10.1371/journal.pone.0129043

**Published:** 2015-06-11

**Authors:** Yi-Ching Tung, Hung-Pin Tu, Wen-Chan Tsai, Cheng-Sheng Chen, Chen-Hsiang Su, Hon-Yi Shi, Chih-Lung Lin

**Affiliations:** 1 Department of Public Health and Environmental Medicine, College of Medicine, Kaohsiung Medical University, Kaohsiung, Taiwan, R.O.C; 2 Departments of Internal Medicine, Kaohsiung Medical University Hospital, Kaohsiung, Taiwan, R.O.C; 3 Departments of Psychiatry, Kaohsiung Medical University Hospital, Kaohsiung, Taiwan, R.O.C; 4 Department of Healthcare Administration and Medical Informatics, Kaohsiung Medical University, Kaohsiung, Taiwan, R.O.C; 5 Department of Neurosurgery, Kaohsiung Medical University Hospital, Kaohsiung, Taiwan, R.O.C; National Taiwan University Hospital, TAIWAN

## Abstract

The aims of this study were to estimate the incidences of herpes zoster (HZ) and postherpetic neuralgia (PHN) in patients after traumatic brain injury (TBI). Furthermore, we aimed to explore the risk factors of the development of HZ and PHN in patients after TBI. This population-based, longitudinal analysis was conducted using the Taiwan National Health Insurance Research Database (consisting of 1,000,000 beneficiaries) from 1996 to 2010. Using the longitudinal National Health Insurance Research Database, we conducted a retrospective population-based cohort study to evaluate the incidence of HZ and PHN in adult TBI patients and controls. Kaplan-Meier analysis and Cox regression were used to compare differences in the development of HZ and PHN. The effects of gender, comorbidity and surgery on the risk of HZ and PHN development were assessed by subgroup analyses. Over a 15-year follow-up, the cumulative incidence of HZ in 28,234 TBI patients (604.00/100,000 person-years) was significantly higher than 34,085 controls (322.21/100,000 person-years) (P<0.0001, by log-rank test). Females showed a significantly higher incidence of HZ than males (p for interaction = 0.0010). The time to HZ development in the follow-up period was 5.9 years in TBI patients compared to 9.9 years in the control set (p <0.0001). TBI patients were 2.93 and 2.11 times likely to develop HZ and PHN, respectively, than the general population. The incidences of HZ and PHN in TBI patients were also significantly greater than for controls in the CCI = 0 subgroup. To our knowledge, this is the first population-based cohort study to reveal that TBI is an independent risk factor for HZ and PHN in TBI patients, especially in females. Physician should pay attention to the possibility of HZ and PHN in TBI patients and be aware that HZ vaccination early after brain trauma may lower the incidence of HZ and PHN.

## Introduction

Herpes zoster (HZ) is a neurocutaneous disease caused by the reactivation of the latent varicella zoster virus (VZV). Direct involvement of the ganglia and the destruction of neurons during VZV reactivation have been suggested in the HZ syndrome. The acute phase is typically signified by neurologic pain that often subsides spontaneously within 2–4 weeks. Complications of HZ include bacterial superinfection and postherpetic neuralgia (PHN) in 20–25% of HZ patients [1–3]. Any complication could increase the cost of HZ-related care, imposing a substantial burden on the health care system [4]. Specific T-cell immunity is responsible for the body’s defense against VZV infection [3]. Declining cellular immunity due to increasing age or immunosuppression has been known to trigger reactivation of VZV [5]. Thus, HZ has been known to occur more frequently in patients with malignancies, human immunodeficiency virus (HIV) infection, transplantation, and immunosuppressive disorders, as well as in those undergoing treatment with immune suppressants [5]. Various diseases associated with impaired immunity, for example, rheumatic and peptic ulcer diseases and malignancies, have been reported to be correlated with an increased risk of HZ [6–8]. However, as many as 90% of HZ cases occur in immunocompetent individuals [9,10]. Although the HZ vaccine, which can reduce the incidence of HZ, has been available since 2006 for adults, the list of high risk groups that the vaccination would be appropriate for has not been clearly defined [11,12]. Thus, successful identification of potential predictors of HZ may help to define high risk patients and assist in the decision-making of administration of HZ vaccinations.

With an annual estimate of 10 million victims affected by new traumatic brain injury (TBI) events, mainly including young adults, TBI is the leading cause of long-term disability and mortality worldwide [13–16]. Considering its impact, TBI is expected to become the third largest cause of global disease burden by 2020 [15]. The incidence of TBI is estimated to be 200–558 per 100,000 people, equaling 1.7 million people in the USA, and the overall economic cost was estimated to be approximately $406 billion USD in 2000 [17–19]. In Taiwan, as many as 52,000 TBIs occur annually, among which, up to 25% are fatal [20,21].

The risk of HZ has been linked to cellular immunity associated with aging and nutrition status [22]. Some postulated mechanisms have been reported to explain the association of TBI with immunosuppression and malnutrition. However, the duration and degree of immunosuppression after TBI are still not clear [23]. Previous research has illustrated that patients with TBI suffer from an increased risk of malnutrition, resulting in altered immunity, which causes the body to be more susceptible to infections [24]. We therefore hypothesized that patients might have a greater risk of developing HZ and PHN after TBI. However, to date, statistical evidence regarding the association between TBI and the incidences of HZ and PHN is limited and therefore worthy of investigation.

The Taiwan National Health Insurance Research Database (NHIRD) is an exceptional database from which we could obtain a representative and reliable calculation of the incidences of HZ and PHN in TBI patients in Taiwan. The aims of this study were to estimate the rates of HZ and PHN in patients following TBI using the NHIRD and to determine whether individuals with TBI were at increased risk for HZ and PHN. Furthermore, we aimed to explore the risk factors in TBI patients for the development of HZ and PHN.

## Methods

### Ethics Statement

The Institutional Review Board of the Kaohsiung Medical University Hospital approved this study in Taiwan. Written consent from study patients was not obtained because the Taiwan Bureau of National Health Insurance (BNHI) is the sole payer in Taiwan and the BNHI dataset consists of de-identified secondary data for research purposes.

### Study design

We conducted a retrospective cohort study from 1996–2010 using the Longitudinal Health Insurance Database (LHID), computed by the Taiwan National Health Insurance program, which contains one million random subjects. The NHIRD releases sets of sampling files for research purposes. The LHID 2010 contains the original claims of 1,000,000 beneficiaries randomly sampled during the period of Jan. 1, 2010 to Dec. 31, 2010. There were no significant differences in the sex, age distribution or insured payroll-related amounts between the patients in the LHID 2010 and those in the original NHIRD.

### Definitions of TBI, HZ and PHN

TBI patients were identified by their associated International Classification of Diseases, 9th revision (ICD-9) codes. The following ICD-9 Clinical Modification (ICD-9-CM) codes were used to identify the patient records in this study: 800.x for a fracture of the vault and base of the skull, 803.x-804.x for other skull fractures, and 850.x-854.x for a concussion, brain contusion, or brain hemorrhage. To exclude very mild TBI patients, TBI patients in outpatient data sets were not included. For the definition of brain surgeries, the relevant procedure codes included craniectomy (01.23, 01.25, 01.39, and 02.01), removal of an epidural hematoma (01.24), removal of an acute subdural hematoma (01.31), removal of a chronic subdural hematoma (01.31), and removal of an intracerebral hematoma (01.39). Exclusion criteria included codes for multiple TBI procedures. Incident HZ cases were identified by an auto-matched search for codes for HZ and HZ complications (ICD-9 code 053.x) present in either an outpatient or inpatient service claim. Patients who were diagnosed with HZ prior to TBI were excluded from the analysis of cumulative incidence. PHN cases were identified by ICD-9 code 053.19. Additionally, we excluded the patients younger than 18 and older than 100 years of age.

Controls were selected using pair matching by age, sex and year of cohort entry in a ratio of approximately 1 to 2 through random selection from the LHID 2010 membership using the PROC SQL procedure of SAS (SAS Institute, Cary, NC). In addition, we also excluded TBI inpatients and healthy subjects who had diagnostic codes in the Charlson Comorbidity Index (CCI) [25] for HIV infection (ICD-9-CM 042.x–044.x), chronic pulmonary disease (416.8, 416.9, 490.x–505.x, 506.4, 508.1, 508.8), rheumatic disease (446.5, 710.0–710.4, 714.0–714.2, 714.8, 725.x), and metastatic solid tumor (196.x–199.x) from the study and control cohorts because these patients may have been treated with immunosuppressive medication or suffered from immunosuppressive conditions that lead to the potential development of HZ [7,26]. Therefore, AIDS/HIV infection, chronic pulmonary disease and metastatic solid tumor were not included in the CCI scores, which included myocardial infarction, congestive heart failure, peripheral vascular disease, cerebrovascular disease, dementia, peptic ulcer disease, mild liver disease, diabetes without chronic complications, diabetes with chronic complications, hemiplegia or paraplegia, renal disease, any malignancy (including lymphoma and leukemia, except for malignant neoplasm of the skin), and moderate or severe liver disease. The CCI scores were subsequently categorized into three levels: 0, 1, and ≥ 2.

### Statistical analysis

Baseline characteristics were compared by t-tests for continuous data and by Pearson’s chi-square test for categorical variables. Incidence was calculated as the number of new cases from 1996 through 2010 divided by the total number of person-years in the available records. We assessed the annual incidence of HZ in all adult TBI patients during the follow-up period. Differences in HZ occurrence between TBI patients and the controls were compared using the log-rank test. Kaplan–Meier analysis was used to calculate the cumulative incidence of HZ between the two groups. Cox proportional hazards models were constructed, and a dichotomous variable denoted whether the patient had HZ or PHN during the study period. Stratified analysis was performed for males and females with HZ after TBI. The covariates used in the models included age, sex and CCI. Interactions between gender and TBI were analyzed using a multiple Cox proportional hazards model with an added interaction term (gender × TBI) and covariates. All statistical analyses were performed using SAS statistical software, version 9.3 (SAS Institute, Cary, NC), and the significance level was set at P < 0.05.

## Results

**Fig 1:**
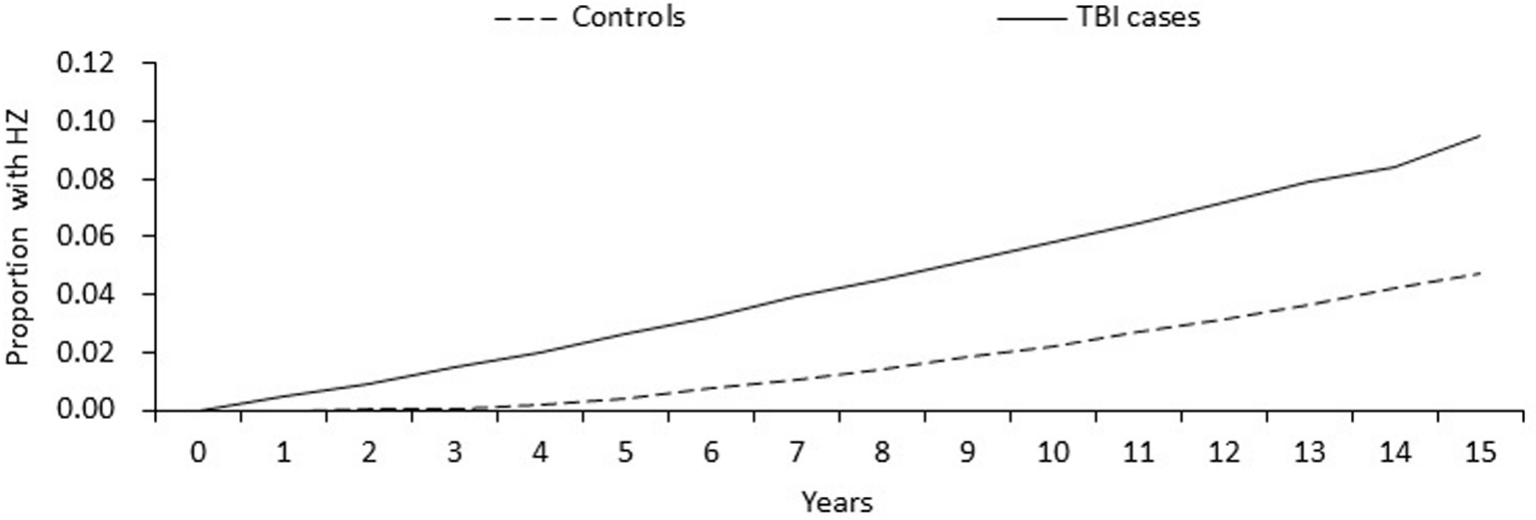
Cumulative incidence of herpes zoster for adult patients with traumatic brain injury and the general population control cohort.

Table 1 shows the patient characteristics in this study. We identified 28,234 adults with TBI and 34,085 controls matched by age and gender from 1996 to 2010. The TBI patients had a lower percentage of CCI scores equal to or greater than 1 based on the dichotomized outcome of the Charlson scores. The HZ development period was significantly faster in the TBI group (5.9 years) compared to the control group (9.9 years) during the follow-up period. The 1-, 5-, 10-, and 15-year actuarial rates of HZ were 0.45%, 2.63%, 5.82%, and 9.53% among TBI patients and 0.00%, 0.43%, 2.23%, and 4.76% among controls, respectively. The cumulative incidence of HZ in TBI patients was significantly higher than that of the control cohorts (Log rank P <0.0001) over the follow-up period (Fig 1).

**Table 1:** Characteristics of adult patients with traumatic brain injury and control cohorts in Taiwan, 1996–2010.

	TBI cases	Controls	P value
	n = 28,234	n = 34,085	
Age (SD), years	45.6 (16.4)	45.7 (15.3)	0.8399
Age group, n (%)			
18–29	5,695 (20.2)	6,268 (18.4)	
30–39	6,497 (23.0)	7,848 (23.0)	
40–49	5,033 (17.8)	6,554 (19.2)	
50–59	5,112 (18.1)	6,528 (19.2)	
60–69	3,232 (11.5)	4,488 (13.2)	
≥70	2,665 (9.4)	2,399 (7.0)	0.5045
TBI-surgical cases, n (%)	1,637 (5.8)		
Gender, n (%)			
Males	16,753 (59.3)	19,991 (58.7)	
Females	11,481 (40.7)	14,094 (41.3)	0.0832
Charlson Comorbidity Index, n (%)			
0	25,729 (91.1)	17,419 (51.1)	
1	1,959 (6.9)	8,092 (23.7)	
≥2	546(1.9)	8,574 (25.2)	<0.0001
Herpes zoster, n (%)	1,466 (5.2)	1,621 (4.8)	<0.0001
Period of developing HZ (SD), years	5.9 (3.6)	9.9 (3.3)	<0.0001

TBI, traumatic brain injury; SD, standard deviation; HZ, herpes zoster

Table 2 shows that the adjusted hazard ratios (HR) for HZ in males and females in the TBI group compared to controls were 2.39 (95% CI 2.13–2.69, p<0.0001) and 2.85 (95% CI 2.50–3.24, p<0.0001), respectively, after adjusting for potential confounders. In addition, the incidence of HZ in TBI was significantly higher in females than in males (P for interaction = 0.0010). The incidence of HZ in TBI patients (604.00 per 100,000 person-years) was significantly greater than that in the control cohort (322.21 per 100,000 person-years). Cox regression revealed that TBI was an independent predictor for HZ after adjusting for age, sex and CCI scores (HR 2.61, 95% CI 2.39–2.85, p<0.0001) (Table 2).

**Table 2:** Incidence and hazard ratios for herpes zoster during the follow-up period for adult patients with traumatic brain injury versus control cohorts.

	HZ	Person-years at risk	Incidence per 100,000 person-years (95% CI)	Adjusted HR (95% CI)	P	P for interaction
Male cohort set						
Controls	891	295,404.64	301.62(282.45–322.09)	1.0		
TBI cases	747	144,931.02	515.42(479.75–553.74)	2.39^a^(2.13–2.69)	<0.0001	
Female cohort set						
Controls	730	207,679.32	351.50(326.91–377.95)	1.00		
TBI cases	719	97,785.88	735.28(683.45–791.04)	2.85^a^(2.50–3.24)	<0.0001	0.19, 0.0010
Total cohort set						
Controls	1,621	503,083.95	322.21(306.90–338.29)	1.00		
TBI cases	1,466	242,716.89	604.00(573.86–635.72)	2.61^b^ (2.39–2.85)	<0.0001	

HZ, herpes zoster; CI, confidence interval; HR, hazard ratio; TBI, traumatic brain injury.

^a^Adjusted HRs with 95% CI and their P values. The results were adjusted for age and Charlson comorbidity index using a Cox proportional-hazards regression model.

^b^Adjusted HRs with 95% CI and their P values. The results were adjusted for age, sex and Charlson comorbidity index using a Cox proportional-hazards regression model.

P for interaction: a Cox proportional-hazards regression model including a gender x TBI interaction was applied

Among the HZ patients, TBI was a significant predictor for PHN after adjusting for age, sex and CCI scores (HR 2.00, 95% CI 1.44–2.76, P<0.0001) in both non-surgical (HR 1.97, 95% CI 1.42–2.74, P<0.0001) and surgical cases (HR 2.36, 95% CI 1.16–4.81, 0.0178) (Table 3). Furthermore, our data suggested that the incidence of HZ in TBI patients, in both nonsurgical and surgical patients, was also significantly greater than in controls in the CCI = 0 subgroup (HR 2.46, 95% CI 2.22–2.72, P<0.0001) or the ≥1 subgroup (HR 2.94, 95% CI 2.48–3.49, P<0.0001) (Table 4). Table 5 shows that the incidence of PHN in TBI patients with HZ was also significantly greater than in controls in the CCI = 0 subgroup (HR 1.67, 95% CI 1.11–2.50, P = 0.0130) or the ≥1 subgroup (HR 2.24, 95% CI 1.33–3.79, P = 0.0026). These results suggest that TBI is associated with an increased risk of developing HZ and PHN, independent of other comorbidities (CCI).

**Table 3:** Incidence and hazard ratios for post-herpetic neuralgia during the follow-up period for adult patients with traumatic brain injury among herpes zoster patients.

	Total	PHN, n (%)	Person-years at risk	Incidence per 100,000 person-years (95% CI)	Adjusted HR (95% CI)	P
HZ without TBI	1,621	88 (5.43)	7,769.57	1,132.62 (919.06–1,395.81)	1.00	
HZ with TBI						
Nonsurgical TBI	1,398	141 (10.09)	5,756.38	2,449.46 (2,076.75–2,889.05)	1.97 (1.42–2.74)	<0.0001
Surgical TBI	68	9 (13.24)	238.64	3,771.34 (1,962.26–7,248.29)	2.36 (1.16–4.81)	0.0178
Combined TBI	1,466	150 (10.23)	5,995.02	2,502.08 (2,132.06–2,936.31)	2.00 (1.44–2.76)	<0.0001

PHN, postherpetic neuralgia; CI, confidence interval; HR, hazard ratio; HZ, herpes zoster; TBI, traumatic brain injury; Adjusted HRs with 95% CI and their P values were adjusted for age, sex and Charlson comorbidity index using a Cox proportional-hazards regression model.

**Table 4:** Incidence and hazard ratios for herpes zoster during the follow-up period for adult patients with traumatic brain injury versus control cohorts with a Charlson comorbidity index = 0 or ≥ 1.

	Total	HZ, n (%)	Person-years at risk	Incidence per 100,000 person-years (95% CI)	HR (95% CI)	P
CCI = 0						
Controls	17,419	647 (3.71)	258,060.22	250.72(232.12–270.80)	1.00	
TBI						
Nonsurgical TBI	24,386	1,247 (5.11)	215,107.94	579.71(548.41–612.80)	2.47 (2.23–2.70)	<0.0001
Surgical TBI	1,343	56 (4.17)	9,447.50	592.75(456.17–770.23)	2.27 (1.72–2.99)	<0.0001
Combined TBI	25,729	1,303 (5.06)	224,555.44	580.26 (549.59–612.64)	2.46 (2.22–2.72)	<0.0001
CCI ≥1						
Controls	16,666	974 (5.84)	245,023.74	397.51(373.32–423.28)	1.00	
TBI						
Nonsurgical TBI	2,211	151 (6.83)	16,577.03	910.90(776.60–1,068.42)	2.96 (2.48–3.53)	<0.0001
Surgical TBI	294	12 (4.08)	1,584.42	757.38(430.12–1,333.63)	2.73 (1.54–4.85)	0.0006
Combined TBI	2,505	163(6.51)	18,161.45	897.51 (769.78–1,046.43)	2.94 (2.48–3.49)	<0.0001

HZ, herpes zoster; CI, confidence interval; HR, hazard ratio; CCI, Charlson comorbidity index; TBI, traumatic brain injury; HRs with a 95% CI and their P values were adjusted for age and sex using a Cox proportional-hazards regression model.

**Table 5:** Incidence and hazard ratios for postherpetic neuralgia during the follow-up period for herpes zoster patients with traumatic brain injury (TBI) versus without TBI with a Charlson comorbidity index = 0 or ≥ 1.

	Total	PHN, n (%)	Person-years at risk	Incidence per 100,000 person-years (95% CI)	HR (95% CI)	P
CCI = 0						
HZ patients without TBI	647	31 (4.79)	3,073.66	1,008.57 (709.29–1,434.13)	1.00	
HZ patients with TBI						
Nonsurgical TBI	1,247	120 (9.62)	5,163.05	2,324.21 (1,943.43–2,779.58)	1.65 (1.10–2.48)	0.0155
Surgical cases TBI	56	9 (16.07)	193.24	4,657.31 (2,423.24–8,951.06)	2.19 (1.00–4.75)	0.0487
Combined TBI	1,303	129 (9.9)	5,356.30	2,408.38 (2,026.65–2,862.01)	1.67 (1.11–2.50)	0.0130
CCI ≥1						
HZ patients without TBI	974	57 (5.85)	4,695.91	1,213.82 (936.29–1,573.63)	1.00	
HZ patients with TBI						
Nonsurgical TBI	151	21 (13.91)	593.33	3,539.38 (2,307.68–5,428.47)	2.41 (1.43–4.05)	0.0009
Surgical TBI	12	0 (0.0)	45.40	-	-	-
Combined TBI	163	21 (12.88)	638.72	3,287.81 (2,143.66–5,042.64)	2.24 (1.33–3.79)	0.0026

PHN, postherpetic neuralgia; CI, confidence interval; HR, hazard ratio; CCI, Charlson comorbidity index; HZ, herpes zoster; TBI, traumatic brain injury; HRs with a 95% CI and their P values were calculated using a Cox proportional-hazards regression model.

The ratios of hazard ratio (RHR) of HZ in controls and TBI patients with a CCI ≥1 compared with a CCI = 0 were 1.36 (95% CI 1.22–1.51, P<0.0001) and 1.11 (95% CI 0.94–1.31, P = 0.2261), respectively (Table 6). Similarly, when we compared patients in the CCI ≥1 and CCI = 0 subgroups, the RHRs of PHN in these HZ patients with and without TBI were not significantly different (Table 6). These data suggested that TBI is a significant prediction factor for either HZ or PHN development, independent of the CCI factor.

**Table 6:** Ratios of hazard ratio for herpes zoster and postherpetic neuralgia for adult patients with a Charlson comorbidity index (CCI) ≥ 1 versus a CCI = 0.

	CCI ≥1 versus CCI = 0	
	RHR (95% CI)	P
HZ		
Controls	1.36 (1.22–1.51)	<0.0001
TBI		
Nonsurgical TBI	1.13 (0.95–1.34)	0.1646
Surgical TBI	0.97 (0.51–1.83)	0.9259
Combined TBI	1.11 (0.94–1.31)	0.2261
PHN		
HZ patients without TBI	1.10 (0.70–1.73)	0.6891
HZ patients with TBI		
Nonsurgical TBI	1.23 (0.77–1.97)	0.3895
Surgical TBI	-	-
Combined TBI	1.09 (0.68–1.73)	0.7298

CCI, Charlson comorbidity index; RHR, ratio of hazard ratio; CI, confidence interval; HZ, herpes zoster; TBI, traumatic brain injury; PHN, postherpetic neuralgia RHRs with a 95% CI and their P values were adjusted for age and sex using a Cox proportional-hazards regression model.

The subgroup of TBI patients with surgical treatment had higher incidences and HRs for HZ (HR 1.05, 95% CI 0.82–1.34, P = 0.1318) and PHN (HR 1.29, 95% CI 0.66–2.54, P = 0.4567) when compared with TBI patients without surgical treatment. However, the differences were not statistically significant (Table 7).

**Table 7:** Incidence and hazard ratios for herpes zoster and postherpetic neuralgia during the follow-up period for surgical versus nonsurgical traumatic brain injury patients.

	Event	Person-years at risk	Incidence per 100,000 person-years (95% CI)	HR (95% CI)	p
Herpes zoster					
Nonsurgical TBI	1,398	231,684.97	603.41(572.59–635.88)	1.00	
Surgical TBI	68	11,031.92	616.39(486.00–781.78)	1.05 (0.82–1.34)	0.1318
Post-herpetic neuralgia					
Nonsurgical TBI	141	5,756.38	2,449.46 (2,076.75–2,889.05)	1.00	
Surgical TBI	9	238.64	3,771.34 (1,962.26–7,248.29)	1.29 (0.66–2.54)	0.4567

CI, confidence interval; HR, hazard ratio; TBI, traumatic brain injury; HRs with a 95% CI and their P values were calculated using a Cox proportional-hazards regression model.

## Discussion

To our knowledge, this is the first population-based cohort study to assess the risk of HZ and PHN in an adult population following TBI. In this study, adults with TBI were found to be at greater risk of developing HZ and PHN than the control cohort. Despite statistical evidence revealing TBI to be the leading cause of morbidity and mortality in the below-45 age group in the industrialized world, TBI is still a growing worldwide medical problem. Globally speaking, the annual incidence rate of HZ ranges from 1.2 to 3.4 among every 1,000 healthy individuals, with a higher rate of 3.9–11.8 among the individuals older than 65 [27–29]. Our results were consistent with a previous report and showed that TBI patients were 2.93 times more likely to develop HZ than the general population with incidences of 604.00 and 322.21 per 100,000 person-years in TBI and control groups, respectively. The adjusted HR of HZ in TBI patients with none of the selected comorbidities was 2.79 compared with the controls (P<0.0001). The data indicate that TBI is an independent risk factor for HZ, and all of the listed confounding effects of comorbidities were excluded.

There has been an active discussion of age and immunosuppression factors [30]. The relationship of these two factors to post-TBI HZ has been excluded by adopting age-adjusted measures (HRs) in this study and by the exclusion of patients with HIV infection, chronic pulmonary disease, rheumatic disease, and metastatic solid tumor as participants.

According to previous studies, women are at higher risk of HZ development in some, but not all, age groups [5]. Our study demonstrated that women have a higher incidence of HZ than men in both the control and TBI groups, with much higher incidence rates in the TBI group (P for interaction = 0.0010). At the same time, the incidences of HZ among women and men in the TBI group were 3.4 and 2.6 times those of women and men in the control group, respectively. However, the information from this study is not sufficient to offer possible explanations for this gender difference in HZ incidence after TBI. Although some possibilities have been mentioned [5], whether women are more vulnerable to HZ than men due to certain psychological, physiological or social differences is worth examining.

The incidence of HZ in the TBI group was significantly higher than that in the control group. Moreover, HZ occurred significantly earlier in the TBI patients than in the controls. Additionally, the growth rate was faster and higher in the TBI patients than in the controls from the beginning until 15 years of the follow-up period. Hence, the risk of HZ should be considered when the treatment of TBI patients is initiated, and preventive measures for HZ should be administered early.

Complications of HZ include bacterial superinfection and PHN in 20–25% of HZ patients [1–3]. Any HZ-related complication could substantially increase the cost and burden of medical care [4]. In the United States, approximately 1 million individuals develop HZ annually, among which 20% of these cases result in PHN [31]. As far as age is concerned, patients with HZ have a lower incidence of PHN in the below-60 age group (less than 10%) compared to the above-60 age group (approximately 40%). Often, the older, more debilitated or immune compromised a population is, the higher its risk of PHN is. In this study, TBI patients with HZ were 2.11 times more likely to develop PHN compared to the general population with HZ. Our results showed that incidences per 100,000 person-years were 1008.57 and 2408.38 in the control and TBI groups, respectively. The lower incidences of PHN in our study may be due to the exclusion of immunocompromised patients in our enrolled cases. The adjusted HRs of PHN in TBI patients with none of the selected comorbidities was 1.67 compared with the controls (P = 0.0130). The data indicate that TBI is associated with an increased risk for developing PHN even after excluding the potential confounding effects of comorbidities.

While considering the impact of comorbidity, patients with a CCI ≥1 had a significantly higher risk, compared with those with a CCI = 0, for the development of HZ, but not for PHN, in the control group. However, regarding both HZ and PHN in the TBI patients, there were no significant differences between the CCI ≥1 and CCI = 0 subgroups. Previous studies have shown that some comorbidities are risk factors of HZ [8]. Our results found the independent impact of TBI on the development of HZ and PHN. When categorizing the TBI group into surgical and non-surgical subgroups, the surgical subgroup was 1.05 and 1.29 times more likely to develop HZ and PHN, respectively, than the non-surgical subgroup. However, the differences were not statistically significant. The small sample size of the subject groups with HZ and PHN undergoing surgical treatment may have caused the lack of statistical significance. Higher CCI in the control group compared to TBI group could be a bias in this study. Thus, the result may underestimate the significance of HZ in the TBI group.

Since the HZ vaccine became available for adults in 2006, and for adults in Taiwan in 2014, its administration has been recommended for immunocompetent individuals aged 60 years or older. However, vaccination rates of zoster vaccine are low [11,12]. There are various studies on the possible risk factors for HZ aimed at producing better strategies for HZ vaccination [6,26]. A recent study by Chen et al. suggested that adults with peptic ulcer disease are at a higher risk for HZ development compared with the general population; therefore, the benefits for HZ vaccination need detailed clinical examination in patients with peptic ulcer disease [8]. Despite the evidence that the HZ vaccine is effective in the prevention of HZ, its effect on PHN is yet to be confirmed. Essentially, if HZ could be prevented, PHZ could also be avoided [29]. Our results showed significantly higher incidences of HZ and PHN in patients following TBI, and the duration of the development of HZ was shorter compared with the control group. We also found that TBI is an independent risk factor for HZ and PHN. Thus, TBI patients have a greater need for vaccination than the general population to lower the incidence of HZ and PHN. In Taiwan, we implemented massive and free varicella vaccination for children in 2014. The impact of this policy on HZ infection in the general population has not yet been observed.

The risk of HZ has been linked to cellular immunity associated with aging and nutrition status [22]. In addition, nutritional deficiencies of vitamin C and zinc have also been identified as predictors for HZ and PHN [32,33]. Possible underlying mechanisms to explain the association of TBI with HZ, as well as with PHN, could be proposed. In an attempt to determine a possible mechanism of immunosuppression in post-TBI patients with HZ, many uncertainties have been demonstrated. First, the CNS has been regarded as an entity that is reactive to peripheral immune infiltration. Nevertheless, recent studies have proven that the opposite may be true, central immunoactivation may impose its effect on the periphery, resulting in peripheral immunological events [34]. Thus various immunologic mediators of the CNS are suggested to be involved in the immune functions and may play a role in the host response to endogenous or exogenous stimuli. Second, in patients suffering from brain injury and undergoing neurosurgery, the release of proinflammatory cytokines into the cerebrospinal fluid may occur with no signs of systemic inflammation, but this has been associated with systemic immunosuppression and an increased risk of infection [35–37]. Third, the early, delayed, and systemic effects of TBI are the result of inflammatory mediators that initiate systemic inflammatory response syndrome (SIRS), resulting in a vicious cycle of hyperinflammation. Consequently, the hypothalamus-pituitary-axis and the sympathetic nervous system provide negative feedback for the hyperinflammation, resulting in compensatory anti-inflammatory response syndrome (CARS). However, in the case of acute TBI, the activation of CARS often causes complications of immunosuppression, such as multi-organ dysfunction syndrome and mortality. Currently, the duration and degree of immunosuppression after TBI are still elusive [23]. Fourth, previous studies have illustrated that patients with TBI suffer from an increased risk of malnutrition, another major factor for immunosuppression. The possible causes are multi-factorial, including poor or imbalanced nutritional intake, decreased mobility, and adverse effects of medication or treatment plans. Subsequently, malnutrition complicates the clinical progression of such patients, hence disturbing the immune system and leaving the body more vulnerable to infections [24]. Other complications of TBI, such as lifelong cognitive, physical and behavioral impairments, may lead to malnutrition, even in patients with mild TBIs [38,39]. In summary, TBI patients are prone to developing HZ and PHN through the possible common pathways of impaired cellular immunity and/or depressed nutritional status after TBI.

Our study is based on non-biased samples from a nationwide population-based database, the NHIRD. With approximately 96% of Taiwan’s population enrolled in the database, the results of our study can be generalized to the population of Taiwan [21].

This study, similar to the situations encountered by studies based on large-scale databases, has the following limitations. First, the clinical picture of TBI, HZ and PHN obtained by analyzing insurance claims data without review of medical records is not as precise as that obtained by analyzing prospective clinical trial data. The difference may be caused by possible errors in the coding of primary diagnoses and treatment modalities in those databases. Moreover, some risk factors could not be obtained through the NHIRD; therefore, the relationship between these factors and the HZ and PHN rates could not be evaluated. Fortunately, given the relative homogeneity of Taiwan’s population, potential confounding factors induced by racial diversity, which may be a risk factor for developing HZ, could be reduced [40]. Nonetheless, future large-scale investigations into other racial groups and geographical regions is needed to determine whether our results may be applicable to the worldwide population [41]. Second, information on nutritional and immunological status of the subjects was not available from our database. Although we adjusted our data for age, sex and comorbidity to validate the negative influence of TBI on the incidence of HZ and PHN in the present study, some unknown confounding factors might still have impacted our results. A further prospective large-scale study is needed to evaluate some unknown confounding factors on the impact of the incidence of HZ and PHN. Third, the Glasgow Coma Scale (GCS) scores and Glasgow Outcome Scale (GOS), which are important factors in evaluating the severity and outcome of TBI, were not included in this study due to their unavailability from the NHIRD database. Therefore, the correlation between the severity and outcome of TBI and the incidences of HZ and PHN could not be established. We found that TBI patients following surgery had a higher tendency to develop HZ and PHN compared with TBI patients without surgery. The patients with surgery were selected from inpatient clinics with moderate to severe TBIs, excluding most of the patients with mild or very severe injuries (GCS of 15 or 3, respectively). Therefore, the severity of the TBI could not be inferred from whether the patient underwent surgery. A further prospective large-scale study is needed to evaluate the impacts of GCS and GOS on the incidence of HZ and PHN.

## Conclusion

To our knowledge, this population based cohort study is the first report to assess the risk of HZ and PHN in an adult population with TBI. In this study, TBI was found to be an independent risk factor for HZ and PHN. The results from our study could draw the attention of physicians to the possibility of HZ and PHN in TBI patients and could bring awareness that early HZ vaccination after brain trauma may be helpful for TBI patients, especially female patients, to lower the incidences of HZ and PHN. Early prevention is the best way to prevent post-herpetic complications, improve the quality of life and reduce the medical burden. The effects of vaccination in TBI patients are worth evaluating. If the mechanism underlying the association between HZ and PHN and TBI could be illustrated in further studies, more effective strategies could hopefully be implemented.
